# Post-malarial neurological syndrome in a Gambian adult residing in the Gambia: a case report and a review of literature

**DOI:** 10.1186/s12936-023-04579-2

**Published:** 2023-05-12

**Authors:** Bertha C. Ekeh, Ebrima Bah, Ya Fatou B M Jobe, Aji F. Daboer, Mariama Gomez, Ridhwan A. Lanlokun

**Affiliations:** 1 Department of Internal Medicine, Edward Francis Small Teaching Hospital, Banjul, The Gambia; 2 Department of Internal Medicine, University of Uyo, Uyo, Nigeria

## Abstract

**Background:**

Post malarial neurological syndrome (PMNS) occurs as a sequel of cerebral malaria which is the most deadly form of severe malaria. In holo-endemic regions (areas of high malarial transmission) all forms of severe malaria as well as cerebral malaria usually occur in children and those who are semi or non-immune like pregnant women, migrants as well as tourists. It also occurs in hypo-endemic regions (areas of limited malarial transmission with low immunity) and malaria- free zones. Survivors however may have neurologic complications after recovery. PMNS has been reported in many parts of the world. Being a sequel to cerebral malaria, it is uncommon in adults who were born and reside in a holo-endemic region all their lives.

**Case report:**

This is the case of an 18 year old Gambian who has lived in The Gambia all his life that had PMNS five days after recovery from cerebral malaria.

**Methods:**

This was a predominantly web based literature search. The search comprise all case reports, original articles and reviews on PMNS or neurological deficits associated with malaria or noted after malaria infection. The search engines used were Google, Yahoo and Google scholar.

**Results:**

A total of 62 papers were found. These were used for this review of the literature.

**Conclusion:**

Cerebral malaria also occurs in adults in holo-endemic areas though rare and some of the survivors may develop PMNS. It is commoner in the youth age group. There is need for further studies since the youth may be a possible new ‘vulnerable group’ in holoendemic areas. This may lead to the widening the targeted group for malaria control in the regions of high malarial transmission.

## Background

Post malaria neurologic syndrome (PMNS) occurs as a sequel to cerebral malaria [1, 2]. It is a rare condition reported from many parts of the world. Features suggesting affectation of different parts of the central and even peripheral nervous system have been documented. The commonest features are features of cerebellar dysfunction which were the earliest symptoms reported [3–11]. Some others include mild to severe encephalopathy, neuropsychiatric features, cranial nerve palsies and more recently Guillain-Barré syndrome. It develops days to about 2 months after recovery from cerebral malaria. At this time, the person is afebrile with disappearance of parasitaemia [2].

The syndrome however is usually self-limiting. Most patients require corticosteroids, and supportive management. PMNS usually occurs as a complication of cerebral malaria though cases of cerebellar ataxia have also been reported after uncomplicated malaria [12–15].

Cerebral malaria is the most severe of all the forms of severe malaria with a high mortality as malaria remains a public health issue [16, 17]. The mortality has decreased significantly with effective anti-malarial medications. Severe malaria usually occurs in children in holoendemic areas, pregnant women or the non-immune [18–20]. Other susceptible population groups are non-immune persons, e.g. tourists or migrant workers moving to holoendemic areas [19–20].

## Case report

The patient is an 18 year old Gambian who has lived in Gambia all his life. He presented with headache, fever and loss of consciousness. He was apparently well until three days prior to presentation when he started having headaches with associated anorexia. There was no associated vomiting, fever, loss of consciousness or seizures. He was able to go to school despite the headaches and fever. He took some analgesics but the headaches and anorexia persisted. A day later he could not be aroused from sleep. He was taken to a peripheral clinic but did not improve after two days. Instead he had two episodes of generalized tonic–clonic seizures. He was therefore rushed to Edward Francis Small Teaching Hospital (EFSTH) for further management.

Examination revealed an acutely ill unconscious boy, afebrile, not pale or jaundiced, nil pitting pedal oedema. There were no rashes. He was unconscious with a GCS of 6/15. Pupils were sluggishly reactive to light. There were no cranial nerve palsies, hemiparesis or features of meningeal irritation. Respiration was grunting, respiratory rate was 24, O_2_ saturation was 98% and there were transmitted sounds all over the chest wall. The examination of other systems was not significant.

The malaria rapid diagnostic test was positive, and the thick film was positive for malaria parasite. Haemoglobin concentration was 11.3 g/dl while the blood group is A Rh positive. The diagnosis was cerebral malaria with aspiration pneumonitis. The White Blood Cell count was 10.2 × 10^9^/L. The differentials showed granulocytes 78.6%, lymphocytes l8.4% and monocytes 3%. The platelet count was 319 × 10/^3^. Electrolytes, urea and creatinine were within normal limits. Aspartate transaminase was elevated to 95.5U/L. Other liver functions were within normal limits.

He was admitted into the ICU. Intravenous artesunate 2.4 mg/Kg was administered at 0 h, 12 h, 24 h then daily for two more days. This was followed up by oral quinine 600 mg three times daily for three days. He also had intravenous ceftriazone 1 g 12-hourly, metronidazole 500 mg 8-hourly and IM metoclopromide. This was followed up with oral doxycycline 100 mg 12 hourly. Seizure was controlled with IV phenytoin 100 mg 12-hourly. Supportive management included oxygen therapy, intravenous fluids, feeding by N/G tube.

He recovered consciousness fully after 3 days. He was therefore transferred back to the main ward since he was fully conscious, was able to walk, eat, speak and take oral medications. Five days later he was noted to be disoriented. There was associated tremors. Examination revealed a young man who was disoriented in time and place but oriented in person. Malaria recrudescence was ruled out since the temperature at this time was 36.9^0^ C. In addition, the thick film performed identified no malarial parasites. He was garrulous with deficits in short term memory, judgment as well as insight. Further examination revealed intention tremors, dysmetria, dysdiadochokinesia, ataxia and impaired tandem walk. A diagnosis of post-malarial neurological syndrome (cerebellar and mild encephalopathy) was made. The relatives requested discharge because of funds considerations. He was discharged on propranolol. The patient however came for follow up about two months later. All the neurological deficits had resolved fully.

## Methods

This was a predominantly web based literature search. The search comprise all case reports, original articles and reviews on PMNS or neurological deficits associated with malaria or noted after malaria infection. The search engines used were Google, yahoo and Google scholar. The electronic source of the databases were PubMed and Medline. In addition snowballing was also used to track down references and citations. This yielded a total of 62 papers which were used in the review.

## Literature review

More than 50 years ago, Lemercier et al. had reported clinical neurologic as well as electroencephalographic abnormalities noted in persons with malaria in Paris in 1969 [3]. However, the earliest documentation of neurological sequelae following malaria was probably by Illangasekera et al. who reported two cases of cerebellar ataxia in Kandy, Sri Lanka in 1976 [4]. The two patients had no fever on presentation but had malaria parasites and were treated with chloroquine and recovered fully. Another early case report of cerebellar ataxia as part of malaria was made in India in 1978 [5]. Thereafter few other reported cases of cerebellar ataxia after malaria [6–11]. In fact amongst these were two case series from Sri Lanka. The first reported 12 cases in 1987[8] while the other reported 8 cases in 1988 [10]. Thereafter, Senanayake and de Silva - the authors who had reported 12 and 8 cases earlier on - carried out prospective studies in two centres in Sri Lanka. They found 74 cases of cerebellar ataxia [21].

Post-malaria neurological syndrome (PMNS) as a term however, was first coined to describe this syndrome in 1996 by Nguyen et al. based on cases from Vietnam and Thailand [1, 2]. The syndrome is defined as the acute onset of neurological or neuropsychiatric symptoms in patients recently recovered from *Plasmodium falciparum* malaria who have negative blood films at the time of onset. This absence of parasitaemia distinguishes it from cerebral malaria, which occurs during parasitaemia. They noted that the time from eradication of the systemic parasitaemia to the development of this syndrome can be from few days to as much as 9 weeks (median time is 4 days) [1, 2].

Nguyen et al. had conducted an observational study in two centres from Vietnam and Thailand In the study, there were a total of 18,124 patients with a diagnosis of falciparum malaria. Those who had severe malaria were 1176. PMNS was seen in some persons who had complicated malaria. They noted that the prevalence of PMNS in patients with *P. falciparum* malaria is 0.12% [1].

The criteria for PMNS diagnosis which they defined were as follows: (1) Recent symptomatic malaria infection with parasites cleared from peripheral blood; (2) In cases of cerebral malaria, full recovery of consciousness; (3) Development of neurological or psychiatric symptoms within two months after the acute illness. The clinical spectrum of PMNS is broad and self-limiting, lasting from 2 to 14 days without any specific treatment [1.2]. PMNS is 300 times more common in patients with severe rather than uncomplicated malaria [1].

Few cases of PMNS have been reported after uncomplicated malaria [12–15]. There was a remarkable case series from Sudan which had 30 patients with post-malarial cerebellar ataxia. Surprisingly, 24(80%) were sequel to uncomplicated malaria while only 6 (20%) were sequel to cerebral malaria [14].

Most cases of PMNS made a complete recovery without specific treatment [2, 12, 22–23].

Two years later, Schnorf et al. classified the syndrome into three subtypes [24]. Their classification is based on clinical severity. These are as follows: (1) A mild and localized encephalopathy affecting the cerebellum and causing isolated cerebellar ataxia or postural tremor (called delayed cerebellar syndrome); (2) A diffuse but not severe encephalopathy causing confusion with or without epileptic seizures; (3) A severe generalized encephalopathy with a good response to steroid therapy [24].

The most common features described included decreased level of consciousness, confusion, fever, generalized seizures, aphasia, tremor, psychosis and myoclonus. Less common manifestations of PMNS include headache, ataxia, weakness, catatonia, acalculia and ophthalmoplegia. The symptoms arise in a variable time after the infection, lasting from 2 up to 60 days after the disappearance of the parasitaemia [11, 24]. They also reported a good response to corticosteroids [24].

Acute Inflammatory Demyelinating Polyneuropathy (AIDP) occurring after malaria though not acknowledged in the classification above has also been reported severally. The earliest report of this seems to have been two cases in India as far back as 1980[25]. More cases have been reported recently [26–35]. One such was a case series that involved 10 patients in Thailand [34]. In a case report from Tunisia, the patient presented with tetraparesis [36]. Bilateral facial nerve palsy which is considered a feature of AIDP has been reported in India [37] and in South Korean [38]. It would therefore seem that AIDP is the fourth subtype of PMNS as noted by one author [29]. The occurrence of Bell’s palsy after *Plasmodium vivax* was reported on a 6 year old in India [23]. In fact the spectrum of PMNS seems to be widening as other neurological deficits are being reported like Parkinson-like features [39] as well as opsoclonus-myoclonus [40].

Neurological deficits after cerebral malaria have been reported in Gambia as far back as 30 years ago [22, 41]. These were prospective studies involving 604 and 624 children, respectively. There were 12% and 23.3% of the survivors who had residual neurological deficits. Cases of PMNS in adults have also reported in The Gambia. However, the patients were Caucasian tourists who came to The Gambia for vacation and had returned to Europe [42, 43]. The most recent was an Italian who was on vacation in The Gambia [43]. All the patients made a full recovery with no long-term neurological sequelae [42].

There are few other reports in the West African region. In a Nigeria report, there were 5 patients with cerebellar ataxia post malarial infection: two children and three adults [44]. The deficits noted were cortical blindness, monoparesis and speech deficits. As at the time of report, a total of five children fully recovered [45]. Another study from Calabar Nigeria, identified 45 children with cerebral malaria. Eleven (28.2%) of the survivors developed neurological sequelae. The commonest deficits were cortical blindness, speech disorders (aphasia or echolalia) and motor abnormalities. Eight of the children recovered fully within 3 weeks. One had persistent hyperactivity till 6 months of age, but developed dyslexia and other learning disabilities till the 3rd year of follow-up. The last two children may have been lost to follow-up [46].

Few cases of PMNS have been reported in adults in Africa. In Sudan, there were 30 patients with post malarial cerebellar ataxia [14]. The ataxia occurred immediately after recovery from malaria. 12 of the patients were treated with prednisolone with consequent full recovery. However, those who had cerebellar infarct or atrophy on CT scan had significantly longer lasting ataxia [14].

Most of the cases of PMNS occurring after malaria infection in adults were actually diagnosed in African migrants who traveled from Africa or persons who vacationed in Africa [1, 15, 42, 43, 47–51]. A French teaching hospital infectious diseases database was investigated for all PMNS cases occurring between 1999 and 2016 and the PubMed database for cases reported by other institutions after 1997. They reported four cases found in the hospital database. All four were Caucasians who had travelled to West Africa. They found 48 other cases who met the case definition in the PubMed database bringing it a total of 52 cases [15].

An 8 year multicentre study which involved 12 countries was carried out in Europe found a total of 185 patients who had severe malaria. Six out of the 46 who had cerebral malaria had neurological deficits at discharge. One had acute cerebellar syndrome which started the third day after malarial treatment [48]. *Plasmodium falciparum* is the deadliest malaria parasite and it is prevalent on the African continent while *P. vivax* is the dominant malarial parasite in most countries outside sub-Saharan Africa. PMNS is commoner after *P. falciparum* infections. However, it is also seen after *P. vivax* infection though much rarer. PMNS was also seen after vivax malaria, notably in children in 50% of the cases and in the Southern Asian countries where *P. vivax* is common [23, 25, 26, 28, 35, 38, 52–55].

Nguyen et al. proposed that Mefloquine was a risk factor for PMNS [1]. However, mefloquine was not administered to most of their patients who had PMNS. Moreover, PMNS also occur after *P. vivax* infection for which mefloquine is not used. These observations cast doubt on an association or causative effect for mefloquine and PMNS [16]. It worthy of note that mefloquine was not administered in most of the cases reported cases including [12, 16, 23, 26, 39, 42, 43, 48–50 and 56]. It is worthy of note that as much as half of the *P. falciparum* patients reported had received a range of treatments (including artemisinin-based combination therapy).

On investigations, it was noted that most patients had hyperparasitaemia during the cerebral malaria phase. This usually clears by the time the diagnosis of PMNS is made. Some recurrent findings include an elevated CSF protein as well as increased cells [42, 47, 49–51]. The cells were mostly lymphocytes and as much as 91% and 100% [50].

Brain CT scan was normal in some cases including [36, 42, 43, 49–51]. In some other cases the CT scan was abnormal. One showed calcified lesions in the temporo-parietal region [52]. MRI was normal in some cases including [23, 42, 49 and 51]. Most of the abnormal cases showed white matter intensities [36, 43, 49, 52–57]. However the sites differed like cerebellar and white matter [47, 49, 52, 53 and 56] periventricular [52, 53] and bilateral hypocampi [57].

In one case, there was a diffuse atrophy of the vermis and both cerebellar hemispheres [56]. The involvement of the white matter seems to be significant in most cases. One case showed diffuse white matter hyper-intensities on T2 weighed images, involving subcortical, deep and 2 periventricular white matter and both external capsule [53]. In the French database that reviewed 52 cases, brain MRIs showed abnormalities in 43% (9/21) of cases with white matter involvement in 100% of the abnormal MRIs [15]. In the case from Tunisia, the brain and spine magnetic resonance imaging (MRI) showed multiple T2-flair hypersignal cerebral lesions predominantly in the white matter of the frontal and the occipital lobes without myelitis or radiculitis [36].

Electroencephalography (EEG) was normal in some cases like [51]. Electroencephalograms were pathological in 93% (14/15) of cases in the French review of the database [15]. EEG showed slowing of waves in one case [52]. In another case, there was a large excess of background activity with frequent runs of high voltage rhythmic slow/sharp activity, in keeping with an encephalopathy [43, 50].

## Discussion

This is the first reported case an adult Gambian indigene who has lived all his life in The Gambia with not just cerebral malaria but also PMNS. The index case was an 18 year old male. This is in keeping with the finding from a recent report in The Gambia, which showed that most persons with cerebral malaria were in the youth age group (15–24) years [58]. It would, therefore, seem that cerebral malaria and the possible sequel PMNS may be commoner in the youths in Gambia. In one case report from France, there two patients who were born in France but of African origin. They presented with cerebral malaria after travel to their indigenous countries, both patients were also 17 and 19 years [50]. The youths who just left the children age group seem to be predisposed to severe malaria. Could it be that there may actually be a paradigm shift in the worse affected age group? Is there a new ‘vulnerable group’ (the youths) for severe malaria and consequent PMNS in the survivors? More prospective studies will be needed to examine this theory. The knowledge that the youths in holoendemic areas of malaria transmission may also be at high risk for severe malaria, cerebral malaria and increased mortality should lead to widening the ‘targeted group’ in malaria control.

It is important to note that some West African countries have reported incidence of severe as well cerebral malaria in indigenous adults who reside in the areas of high malaria transmission [59–61]. The reported case from Nigeria where cerebral malaria was misdiagnosed as hepatic encephalopathy and correct diagnosis only made at autopsy is worthy of note[62]. This will suggest that cerebral malaria may actually be underdiagnosed or misdiagnosed in some West African countries. By inference, there may also be many cases of misdiagnosis of PMNS. Physicians in the endemic areas like West Africa need to have a high index of suspicion in all adults especially the youths presenting with fever, seizures and loss of consciousness as this may be cerebral malaria. There is also a need to reevaluate such patients for features of PMNS.

The index patient presented with features of both acute encephalopathy as well as cerebral ataxia five days after he recovered from cerebral malaria; he fits the case definition by Nguyen et al. [1]. He was disoriented, garrulous with impairment in judgment and insight. He also had tremors, ataxia, dysmetria and dysdiadochokinesia. His features are in keeping with the first subtype and partly the third subtype as classified by Schnorf et al. [24]. In most cases, the cerebellar ataxia was noted to be delayed [6, 8, 21, 47 and 56]. However, this patient had features of cerebellar ataxia which appeared five days after he regained consciousness. This is similar to one report from Europe [48]. The index patient was treated with artesunate and thereafter oral quinine giving more credibility that mefloquine is not likely a risk or an aetiologic factor in PMNS. The patient came for follow up 2 months later. He had recovered fully as reported in most other cases.

## Conclusion

Cerebral malaria is considered a rare entity in the African adult who resides fully in Africa. Hence PMNS which is a rare complication is even rarer. In view of this, many cases of PMNS have not been reported in African adults who fully reside in Africa. This is one of the earliest cases and the authors hope that it serves to create awareness. This will consequently lead to a high index of suspicion. There is also need to examine the possibility of the youths as being the new vulnerable group in holoendemic areas for severe malaria. In view of this the World Health Organization may widen the ‘targeted group’ for interventions for the control of malaria.

## Data Availability

Further information is available from the corresponding author on reasonable request as no confidential information will be disclosed.
